# Glucocorticoid Modulates Angiotensin II Receptor Expression Patterns and Protects the Heart from Ischemia and Reperfusion Injury

**DOI:** 10.1371/journal.pone.0106827

**Published:** 2014-09-29

**Authors:** Qin Xue, Andrew J. Patterson, Daliao Xiao, Lubo Zhang

**Affiliations:** Center for Perinatal Biology, Division of Pharmacology, Department of Basic Sciences, Loma Linda University School of Medicine, Loma Linda, California, United States of America; Indiana University School of Medicine, United States of America

## Abstract

Glucocorticoid regulates angiotensin II receptor (ATR) expression *via* activating glucocorticoid receptors and binding to glucocorticoid response elements. The regulation of ATR by glucocorticoids in the context of myocardial injury from ischemia/reperfusion (I/R) is yet to be elucidated. The present study determined the role of ATR in glucocorticoid-induced cardiac protection. Adult male rats were administered once a day *i.p.* 1 mg/kg/day dexamethasone or dexamethasone plus 10 mg/kg/day RU486 for 5 days. Hearts were then isolated and subjected to I/R injury in a Langendorff preparation. Dexamethasone treatment significantly decreased I/R injury and improved post-ischemic recovery of cardiac function. Dexamethasone increased glucocorticoid receptor binding to glucocorticoid response elements at AT_1a_R and AT_2_R promoters, resulting in a significant increase in expression of AT_1_R protein but a decrease in AT_2_R expression in the heart. In addition, dexamethasone treatment significantly increased PKCε expression and p-PKCε protein abundance. These dexamethasone-mediated effects were blocked by RU486. More importantly, blockade of AT_1_R and AT_2_R with losartan and PD123319 abrogated dexamethasone-induced protection of the heart from I/R injury. The results indicate that glucocorticoid promotes a cardioprotective phenotype associated with the upregulation of AT_1_R and PKCε and downregulation of AT_2_R in the heart.

## Introduction

Glucocorticoids play a vital role in systemic stress response as well as in regulating vascular integrity, inflammatory and immune response through the regulation of cellular homeostasis and gene activity [1]–[3]. It is well established that glucocorticoids are beneficial in a variety of disease states [4]. There is increasing interest in glucocorticoid therapy in non-autoimmune and non-inflammatory diseases such as acute myocardial infarction, angina, cardiac amyloidosis as well as cardiopulmonary-bypass surgery [5]. Whereas prenatal glucocorticoid treatment was associated with cardiac dysfunction and myocardial remodeling later in life [6], short-term glucocorticoid treatment of adult rats for 1 to 10 days showed a cardioprotective effect in myocardial ischemia and reperfusion injury [7]–[9]. The exact pathway by which glucocorticoids achieve the protection is not clear, but studies suggest that glucocorticoids decrease proapoptotic factors such as p38/MAPK, cytochrome C and increase prosurvival genes HSP72 [7]–[9]. Glucocorticoids regulate genomic activity through the glucocorticoid receptor (GR), a ligand-dependent transcription factor that activates or represses gene transcription through binding to glucocorticoid responsive element (GRE) in promoters of target genes [10].

Angiotensin II (Ang II) plays a key role in the cardiovascular homeostasis. In addition to maintaining normal vascular integrity, Ang II is involved in disease progression in conditions such as hypertension, myocardial remodeling with ventricular hypertrophy, heart failure and ischemic heart disease [11]–[14]. Ang II acts on two main G-protein-coupled receptor subtypes: type 1 (AT_1_R) and type 2 (AT_2_R) Ang II receptor [15]. Upregulation of AT_1_R in the acute setting is associated with cardioprotection from ischemia reperfusion injury, whereas AT_2_R is associated with increased myocardial injury [15]. Activation of AT_1_R promotes cell hypertrophy and proliferation, while activation of AT_2_R counteracts AT_1_R-mediated effects and induces apoptosis [16], [17]. The expression of AT_1_R and AT_2_R are regulated by glucocorticoids [16]. Several GREs have been identified in rat AT_1a_R and AT_2_R promoters [18]–[20]. Stimulation of GREs on AT_1a_R promoter increases gene activity while GREs at AT_2_R represses promoter activity.

The effect of glucocorticoids on the expression profile of ATRs in the heart and cardiac recovery from ischemic reperfusion insult is not clear. The present study tests the hypothesis that dexamethasone treatment protects rat heart function from global ischemia and reperfusion injury and that the mechanism of this protection involves GR-mediated fine-tuning of ATR expression patterns in the heart. Herein we present evidence that dexamethasone induces the protection in rat hearts from ischemia/reperfusion-mediated injury associated with increasing the expression of AT_1_R and decreasing AT_2_R. We further demonstrate that dexamethasone regulates ATR expression and protects the heart through a GR dependent mechanism.

## Materials and Methods

### 1. Experimental animals

All procedures and protocols were approved by the Institutional Animal Care and Use Committee of Loma Linda University and followed the guidelines by National Institutes of Health Guide for the Care and Use of Laboratory Animals. All animals undergoing surgical procedures were anesthetized with ketamine and xylazine (87 mg/kg and 13 mg/kg), and adequate anesthesia was determined by loss of pedal withdrawal reflex. Three-month-old male Sprague-Dawley rats were purchased from Charles River Laboratories (Portage, MI). Rats were injected intraperitoneally with either 1 mg/kg/day dexamethasone for 5 days or vehicle control. An additional group of rats was injected intraperitoneally with 10 mg/kg/day RU486 1 hour before the dexamethasone injection.

### 2. Hearts subjected to ischemia and reperfusion

Hearts were isolated and perfused in retrograde *via* the aorta in a modified Langendorff apparatus, as previously described [20]. After the baseline recording, hearts were perfused in the absence or presence of losartan (1 µM, a selective AT_1_R antagonist) and/or PD123319 (0.3 µM, a selective AT_2_R antagonist) 5 minutes before ischemia/reperfusion, followed by subjection to 20 minutes of global ischemia and 30 minutes of reperfusion. The blockers were continuously present in the heart perfusion solution during the ischemia/reperfusion period. Left ventricular developed pressure (LVDP), heart rate (HR), dP/dt_max_, dP/dt_min_, and LV end diastolic pressure (LVEDP) were continuously recorded. Myocardial infarct size was measured as described previously [20]. Briefly, left ventricles were collected after 60 minutes of reperfusion, cut into four slices, incubated in 1% triphenyltetrazolium chloride for 15 minutes at 37°C, and immersed in formalin for 30 minutes. Each slice was then photographed (Kodak digital camera) separately, and the areas of myocardial infarction in each slice were analyzed by computerized planimetry (Image-Pro Plus), corrected for the tissue weight, summed for each heart, and expressed as a percentage of the total left ventricular weight. Lactate dehydrogenase (LDH) activity was measured in coronary effluent collected at 30 seconds before the onset of ischemia, and at 0, 1, 2, 3, 4, 5, 10, 15, 20, and 30 minutes of reperfusion. LDH activity was measured using a standard assay (TOX 7 kit, Sigma, Saint Louis, MO), following the manufacture's directions [20]. In brief, the lactate dehydrogenase assay mixture was prepared by mixing equal volumes of LDH assay substrate solution, LDH assay dye solution, and 1 × LDH assay cofactor preparation provided by the kit. The LDH assay mixture was then added to each sample in a volume ratio of 2∶1. The sample mixture was protected from light and incubated at room temperature for 30 minutes. The reaction was terminated by the addition of 1/10 volume of 1N HCl. The absorbance was measured at a wavelength of 490 nm. According to the standard curve, LDH was analyzed and expressed as area under the curve (AUC).

### 3. Western blot analysis

For the studies of Western blotting, qRT-PCR, electrophoretic mobility shift assay and chromatin immunoprecipitation, hearts were isolated from animals treated with vehicle control, dexamethasone or dexamethasone plus RU486, but not subjected to ischemia and reperfusion treatments. Left ventricles were collected. Tissues were homogenized in a lysis buffer containing (in mM) 150 NaCl, 50 Tris.HCl, 10 EDTA, 0.1 phenylmethylsulfonyl fluoride, 0.1% Tween-20, 1% Triton, 0.1% β-mercaptoethanol, 5 µg/ml leupeptin, and 5 µg/ml aprotinin, pH 7.4, and allowed to incubate for 1 hour on ice. Homogenates were then centrifuged at 4°C for 10 minutes at 10,000 g, and supernatants collected. Nuclear extracts were prepared from hearts using NXTRACT CelLytic Nuclear Extraction Kit (Sigma). Protein concentrations were measured using a protein assay kit (Bio-Rad, Hercules, CA). Samples with equal amounts of protein were loaded onto 10% polyacrylamide gel with 0.1% SDS and separated by electrophoresis at 100 V for 90 minutes. Proteins were then transferred onto nitrocellulose membranes. Nonspecific binding sites was blocked for 1 hour at room temperature in a Tris-buffered saline solution containing 5% dry-milk. The membranes were then probed with primary antibodies against PKCε (1∶500 dilution), PKCδ (1∶500 dilution) (Santa Cruz Biotechnology; Santa Cruz, CA), phospho- PKCε (1∶500 dilution), phospho-PKCδ (1∶500 dilution) (Millipore, Billerica, MA), AT_1_R (1∶100 dilution), AT_2_R (1∶200 dilution), glucocorticoid receptor (GR, 1∶2000 dilution) (Santa Cruze). To assure equal loading, band intensities were normalized to actin. After washing, membranes were incubated with secondary horseradish peroxidase-conjugated antibodies. Proteins were visualized with enhanced chemiluminescence reagents, and blots were exposed to Hyperfilm. The results were analyzed with the Kodak ID image analysis software.

### 4. Real-time RT-PCR

RNA was extracted from left ventricles using TRIzol protocol (Invitrogen, Carlsbad, USA). PKCε, PKCδ, AT_1a_R, AT_1b_R and AT_2_R mRNA abundance was determined by real-time RT-PCR using Icycler Thermal cycler (Bio-Rad), as described previously [20]. The primers used were: PKCε, 5′-gctcaagggtaaggatgaagt-3′ (forward) and 5′-ctggaagcagcaatagagttg-3′ (reverse); PKCδ, 5′-acagaagaagcccaccat-3′ (forward) and 5′-gaactcagccttcccgtt-3′ (reverse); AT_1a_R, 5′-ggagaggattcgtggcttgag-3′ (forward) and 5′-ctttctgggagggttgtgtgat-3′ (reverse); AT_1b_R_,_ 5′-atgtctccagtcccctctca-3′ (forward) and 5′-tgacctcccatctccttttg-3′ (reverse); and AT_2_R, 5′-caatctggctgtggctgactt-3′ (forward) and 5′-tgcacatcacaggtccaaaga-3′ (reverse). Real-time RT-PCR was performed in a final volume of 25 µl. Each PCR reaction mixture consisted of primers, iScript reverse transcriptase and iQ SYBR Green Supermix (Bio-Rad). We used the following RT-PCR protocol: 50°C for 10 minutes, 95°C for 5 minutes, followed by 40 cycles of 95°C for 10 seconds, 56°C for 30 seconds, 72°C for 20 seconds. The reaction efficiencies of primers were in the range of 95 to 99%. GAPDH was used as an internal reference and serial dilutions of the positive control were performed on each plate to create a standard curve. PCR was performed in triplicate, and threshold cycle numbers were averaged.

### 5. Electrophoretic mobility shift assay (EMSA)

Nuclear extracts were prepared from left ventricles using NXTRACT CelLytic Nuclear Extraction Kit (Sigma). The oligonucleotide probes of GREs at rat AT_1a_R and AT_2_R promoter region were labeled and subjected to gel shift assays using the Biotin 3′ end labeling kit and LightShift Chemiluminescent EMSA Kit (Pierce Biotechnology, Rockford, IL), as previously described [20], [21]. Briefly, single stranded oligos were incubated with Terminal Deoxynucleotidyl Transferase (TdT) and Biotin-11-dUTP in binding mixture for 30 minutes at 37°C. The TdT adds a biotin labeled dUTP to the 3′-end of the oligonucleotides. The oligos were extracted using chloroform and isoamyl alcohol to remove the enzyme and unincorporated biotin-11-dUTP. Dot blots were performed to ensure the oligos were labeled equally. Combining sense and antisense oligos exposing to 95°C for 5 minutes was done to anneal complementary oligos. The labeled oligonucleotides were then incubated with or without nuclear extracts in the binding buffer (from LightShift kit). Binding reactions were performed in 20 µl containing 50 fmol oligonucleotieds probes, 1× binding buffer, 1 µg of poly (dI-dC), and 5 µg of nuclear extracts. For competitions studies, increasing concentrations of non-labeled homologous and heterologous oligonucleotides were added to binding reactions. The samples were then run on a native 5% polyacrylamide gel. The contents of the gel were then transferred to a nylon membrane (Pierce, Rockford, IL) and crosslinked to the membrane using a UV crosslinker (125 mJoules/cm^2^). Membranes were blocked and then visualized using the reagents provided in the LightShift kit.

### 6. Chromatin immunoprecipitation (ChIP)

Chromatin extracts were prepared from left ventricles. ChIP assays were performed using the ChIP-IT kit (Active Motif, Carlsbad, CA), as previously described [22]. Briefly, hearts were exposed to 1% formaldehyde for 10 minutes to crosslink and maintain DNA/protein interactions. After the reactions were stopped with glycine, hearts were washed, chromatin isolated and the DNA sheared into fragments (100 – 1000 base pairs) using a sonicator. ChIP reactions were performed using GR antibody to precipitate the transcription factor/DNA complex. Crosslinking was then reversed using a salt solution and proteins digested with proteinase K. Primers flanking GRE1, GRE2, GRE3 at the promoter of AT_1a_R were used: 5′-tggaaccaatgctgcttgtaa-3′ (forward) and 5′-cgagtcctcactagcaattca-3′ (reverse); 5′-ttgctagtgaggactcgaatc-3′ (forward) and 5′-gcatcgggagccagaatca-3′ (reverse); 5′-atgccatctgtaatccacaac-3′ (forward) and 5′-gccacatgaactgactccaa-3′ (reverse). Primers flanking GRE4, GRE5, GRE6, GRE7, GRE8 at the promoter of AT_2_R were used: 5′-gcaagcagggtagagattaaa-3′ (forward) and 5′-gacagattttaaataaattccc-3′ (reverse); 5′-tgagtaactaatatccccattt-3′ (forward) and 5′-gcttcacaagccacatctca-3′ (reverse); 5′-gct gctgctggctggtat-3′ (forward) and 5′-acaagtaaggatgattatg-3′ (reverse); 5′-ttaatgttttgcagccagaaa-3′ (forward) and 5′-ggggagccttcaacctacat-3′ (reverse); 5′-ccagaggtct ggtgcagtta-3′ (forward) and 5-acttaccttaaaatgcaggct-3′ (reverse). PCR amplification products were visualized on 2% agarose gel stained with ethidium bromide. To quantify PCR amplification, Real-time PCR were carried out with 5 minutes initial denaturation followed by 45 cycles of 95°C for 10 seconds, 56°C for 30 seconds, and 72°C for 10 seconds, using the iQ SYBR Green Supermix with iCycler real-time PCR system (Bio-Rad).

### 7. Statistical analysis

Data are expressed as mean ± SEM. Statistical significance (*P*<0.05) was determined by analysis of variance (ANOVA) followed by Neuman-Keuls post hoc testing or Student's *t* test, where appropriate.

## Results

### 1. Dexamethasone up-regulated AT_1_R and down-regulated AT_2_R in the heart

**Figure 1:**
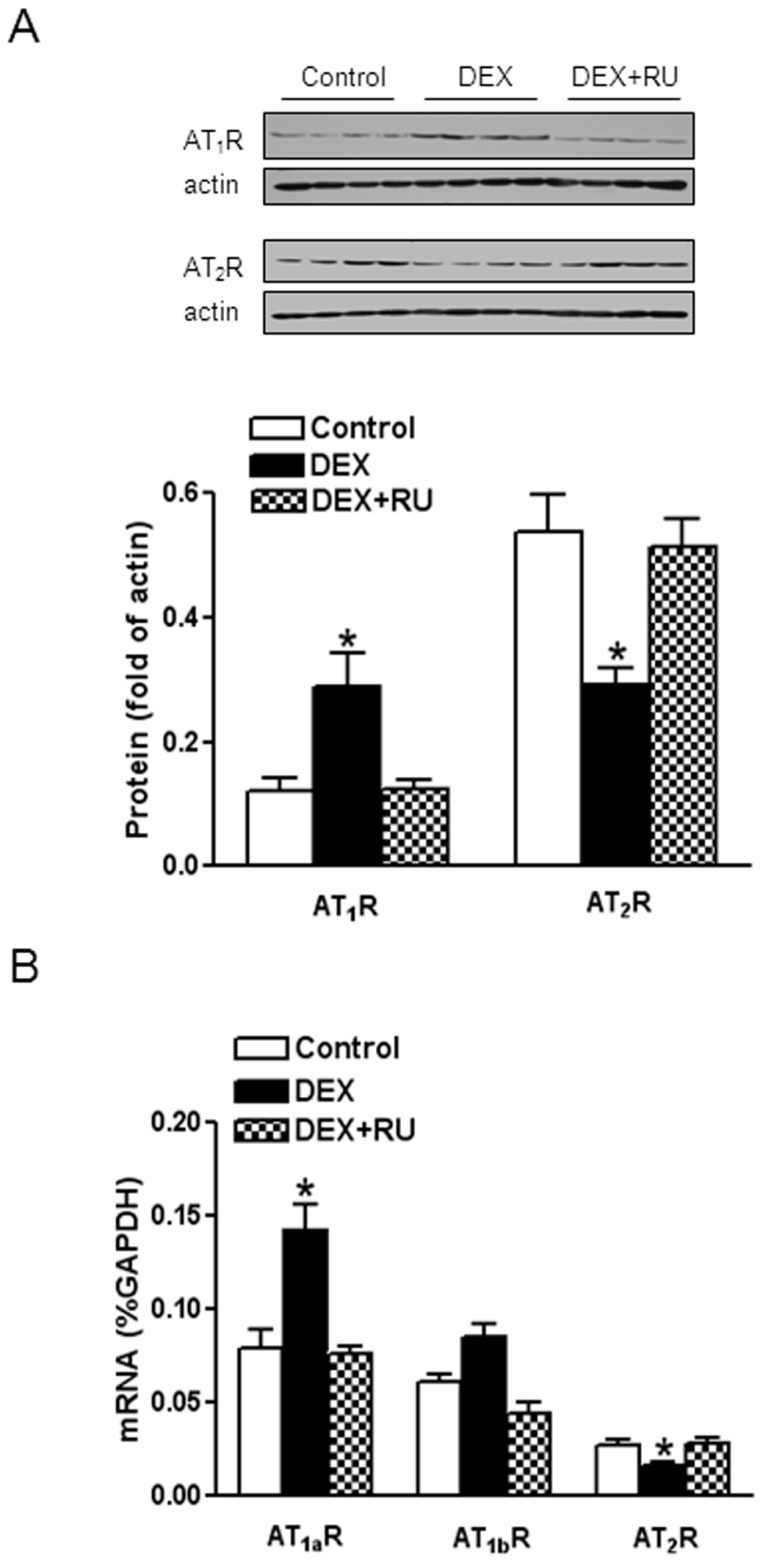
Effects of dexamethasone on AT_1_R and AT_2_R mRNA and protein. Hearts were isolated from animals treated with vehicle control, dexamethasone (DEX), DEX + RU486 (RU). AT_1_R and AT_2_R protein (**A**) and mRNA (**B**) abundance was determined by Western blots and real time RT-PCR, respectively. Data are means ± SEM. Data were analyzed by one-way ANOVA. * P<0.05, DEX *vs*. control or DEX+RU486, n = 4.

Dexamethasone treatment significantly increased AT_1_R protein abundance but decreased AT_2_R abundance in the left ventricle (Figure 1A). Unlike large mammals including humans in which only one type of AT_1_R is identified, rodents have two subtypes of AT_1_R, AT_1a_R and AT_1b_R from two separate genes. Whereas AT_1a_R is a human equivalent AT_1_R, AT_1a_R and AT_1b_R are functionally and pharmacologically indistinguishable at the protein level. Dexamethasone significantly increased AT_1a_R mRNA and decrease AT_2_R mRNA, but had no significant effect on AT_1b_R mRNA (Figure 1B). A GR inhibitor RU486 blocked dexamethasone-induced up-regulation of AT_1_R and down-regulation of AT_2_R gene expression in the heart (Figure 1).

### 2. Dexamethasone protected the heart from ischemia and reperfusion injury

**Figure 2:**
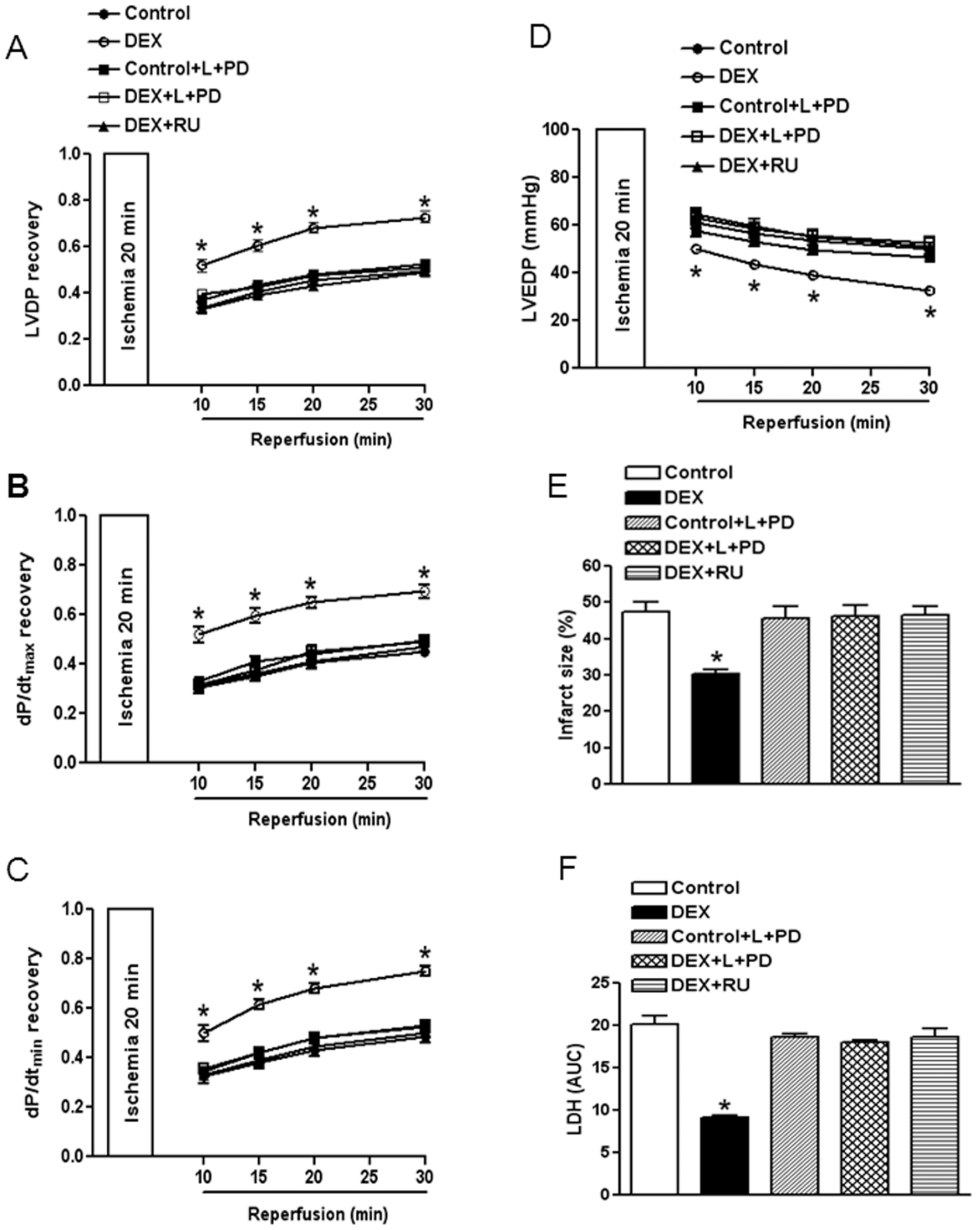
Effects of dexamethasone on cardiac ischemia and reperfusion injury. Hearts were isolated from animals treated with vehicle control, dexamethasone (DEX), DEX + RU486 (RU), and were subjected to 20 minutes of ischemia and 30 minutes of reperfusion injury in a Langendorff preparation. Losartan (L, 1 µM) and PD123319 (PD, 0.3 µM) were added 5 minutes before ischemia and continuously present in the perfusion buffer during ischemia/reperfusion period. Post-ischemic recovery of left ventricular function and infarct size were determined. LDH release over 30 minutes of reperfusion was measured as area under curve (AUC). Data are means ± SEM. Data were analyzed by two-way ANOVA. * P<0.05, DEX *vs*. control or DEX+RU486, n = 6.

**Figure 3:**
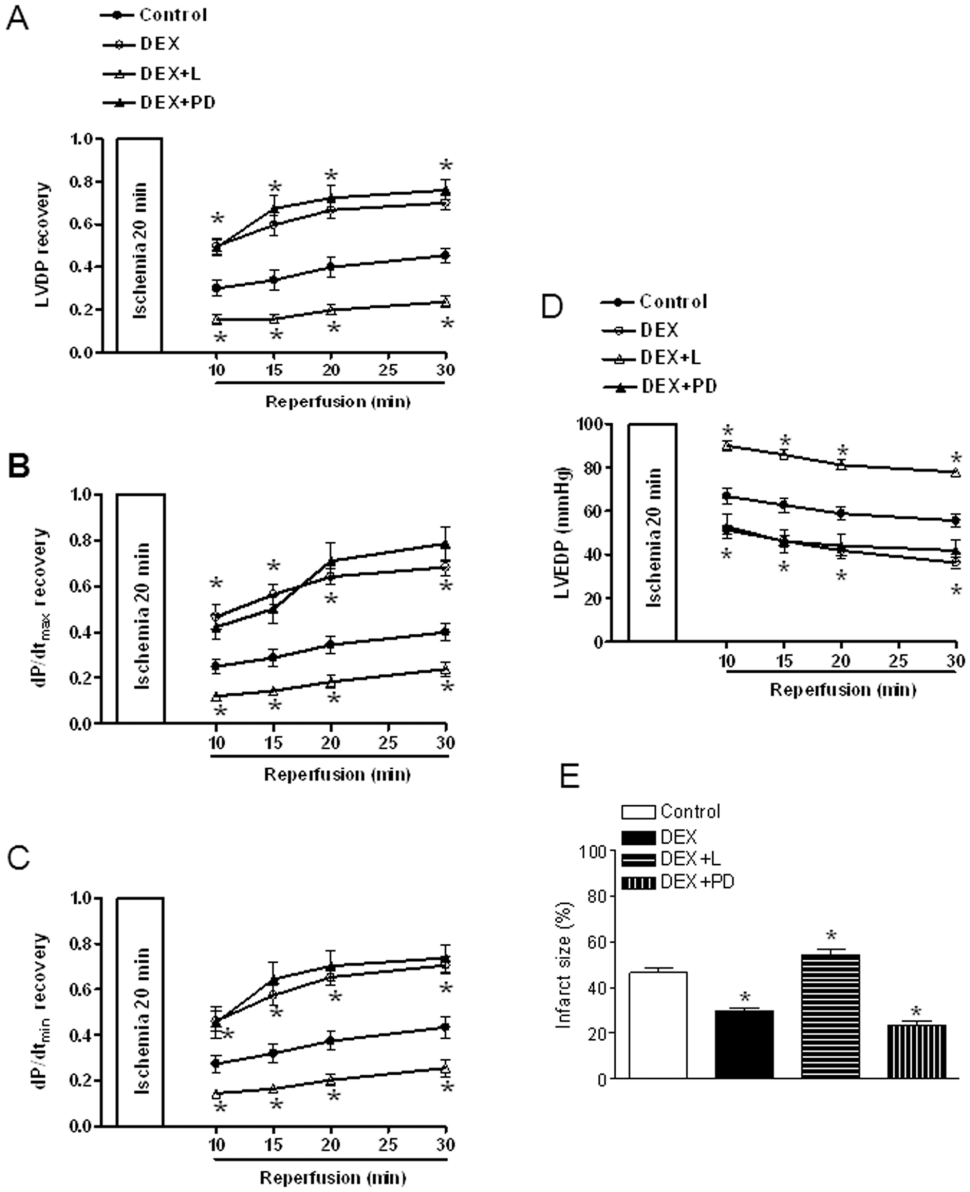
Effects of individual application of losartan and PD123319 on dexamethasone-induced cardiac protective effect. Hearts were isolated from animals treated with vehicle control or dexamethasone (DEX) and were subjected to 20 minutes of ischemia and 30 minutes of reperfusion injury in a Langendorff preparation. Losartan (L, 1 µM) or PD123319 (PD, 0.3 µM) were added 5 minutes before ischemia and continuously present in the perfusion buffer during ischemia/reperfusion period. Post-ischemic recovery of left ventricular function and infarct size were determined. Data are means ± SEM. Data were analyzed by two-way ANOVA. * P<0.05, DEX *vs*. control or DEX+L, n = 4-9.

Dexamethasone treatment had no significant effect on baseline cardiac parameters (Table 1), but significantly improved the recovery of myocardial function by increasing LVDP, dP/dt_max_ and dP/dt_min_ after 20 minutes of global ischemia followed by 30 minutes of reperfusion (Figure 2A, 2B, 2C). Consistent with these findings, dexamethasone reduced ischemia and reperfusion-induced myocardial injury by decreasing LVEDP, myocardial infarct size and LDH release (Figure 2D, 2E, 2F). The results of myocardial infarct size determined at 60 minutes of reperfusion were supplemented and supported with quantitative data of cardiac injury by the measurement of lactate dehydrogenase (LDH) release from perfusates, as shown in Figure 2E and 2F. These effects were abrogated by RU486 (Figure 2). We further determined the functional significance of dexamethasone-induced increase in AT_1_R and decrease in AT_2_R expression in glucocorticoid-mediated cardiac protection by blocking both AT_1_R and AT_2_R with losartan and PD123319. As shown in Figure 2, in the presence of losartan and PD123319, the dexamethasone-induced protective effect in the heart was abrogated. In addition, our previous study demonstrated a context-specific function of both AT_1_R and AT_2_R in the acute setting of myocardial ischemia and reperfusion injury in rats [20]. To distinguish the function of AT_1_R and AT_2_R on glucocorticoid-mediated protection in the heart subjected to I/R, we further determined the effect of individual application of AT_1_R and AT_2_R blockers on dexamethasone-induced cardiac protection. As shown in Figure 3, losartan alone, but not PD123319, abrogated the dexamethasone-induced protective effect in the heart.

**Table 1:** Pre-ischemic left ventricular functional parameters.

Group	HR (bpm)	LVEDP (mmHg)	LVDP (mmHg)	dP/dt_max_ (mmHg/s)	dP/dt_min_ (mmHg/s)	CF (ml/min)
C	268±4.4	5.1±0.2	107±2.2	3694±107.4	2278±100.3	10.8±0.5
DEX	252±6.5	4.8±0.2	112±2.2	3729±114.1	2314±57.0	12.0±0.4
C+L+PD	256±4.5	4.8±0.2	103±3.9	3718±172.6	2307±92.8	10.8±0.4
DEX+L+PD	260±4.7	4.8±0.1	115±2.4	3818±92.5	2378±48.0	11.4±0.4
DEX+RU	257±2.9	5.1±0.2	109±2.7	3643±34.8	2255±39.7	12.3±0.4

HR, heart Rate; LVDP, left ventricular developed pressure; LVEDP, left ventricular end diastolic pressure; dP/dt_max_, maximal rate of contraction; dP/dt_min_, maximal rate of relaxation; CF, coronary flow; C, control; DEX, dexamethasone; RU, RU486; L, losartan; PD, PD123319; n = 5.

### 3. Dexamethasone increased GR binding to GREs at AT_1a_R and AT_2_R promoters

**Figure 4:**
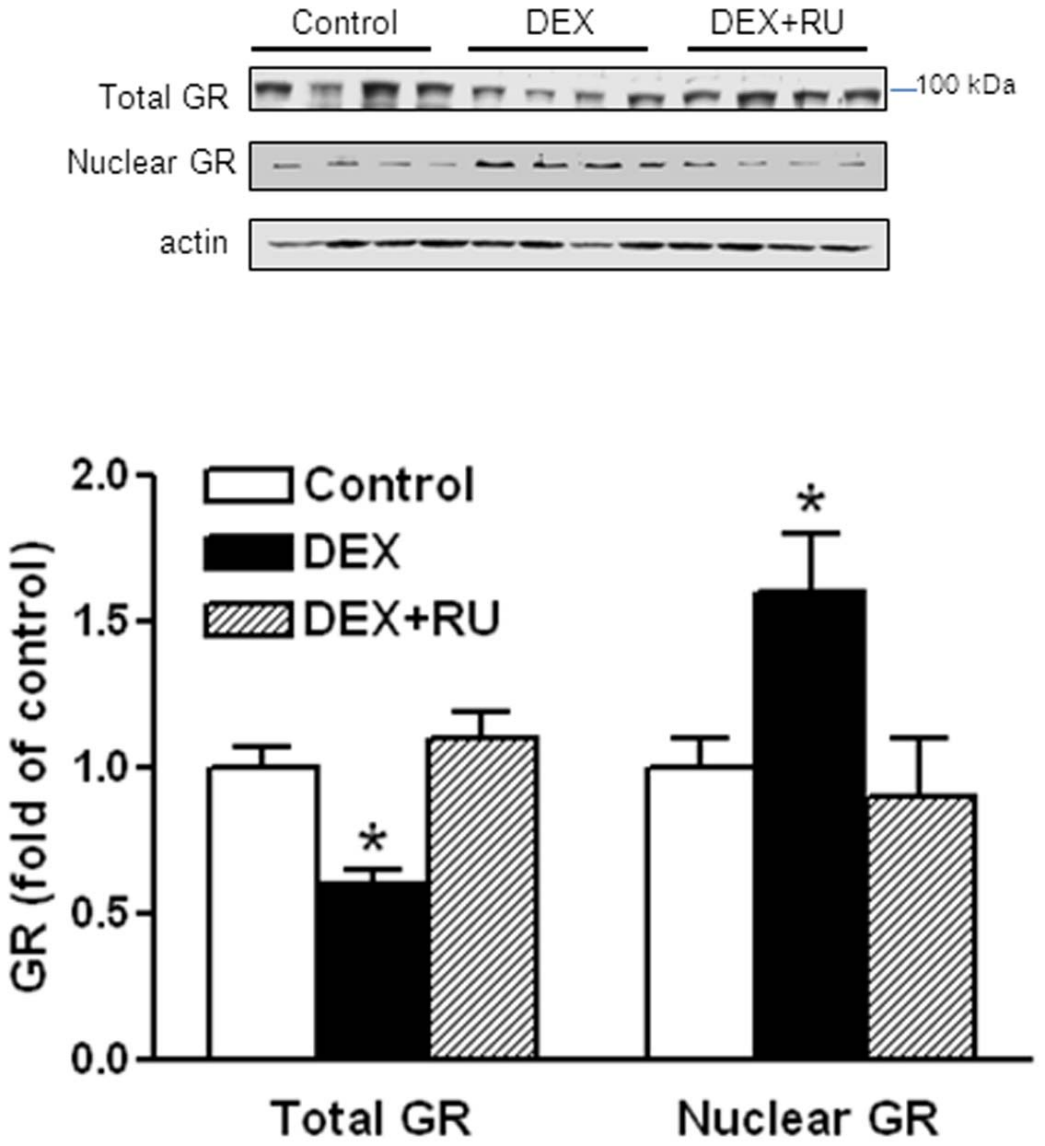
Effects of dexamethasone on GR protein abundance. Hearts were isolated from animals treated with vehicle control, dexamethasone (DEX), DEX + RU486 (RU). Total and nuclear GR protein abundance was determined by Western blots. Data are means ± SEM. Data were analyzed by one-way ANOVA. * P<0.05, DEX *vs*. control or DEX+RU486, n = 4.

**Figure 5:**
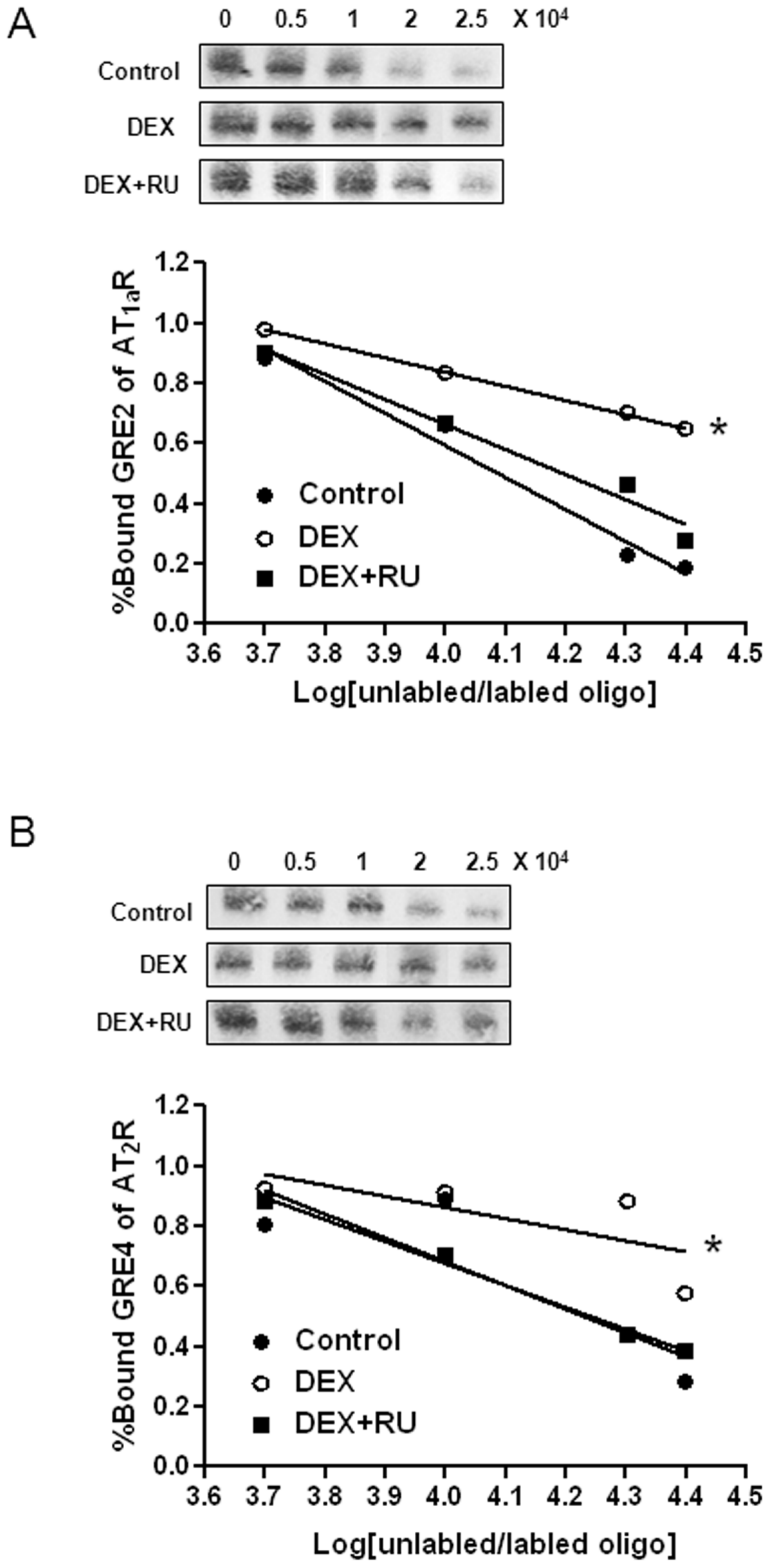
Effects of dexamethasone on GR binding affinity to GREs at the AT_1a_R and AT_2_R promoters. Hearts were isolated from animals treated with vehicle control, dexamethasone (DEX), DEX + RU486 (RU). Competition EMSA was performed with pooled nuclear extracts (n = 5) with the increasing ratio of unlabeled/labeled oligonucleotides encompassing the GRE2 at the AT_1a_R promoter (**A**) or the GRE4 at the AT_2_R promoter (**B**). Data are means ± SEM. The slopes were analyzed by one-way ANOVA. * P<0.05, DEX *vs*. control or DEX+RU486.

**Figure 6:**
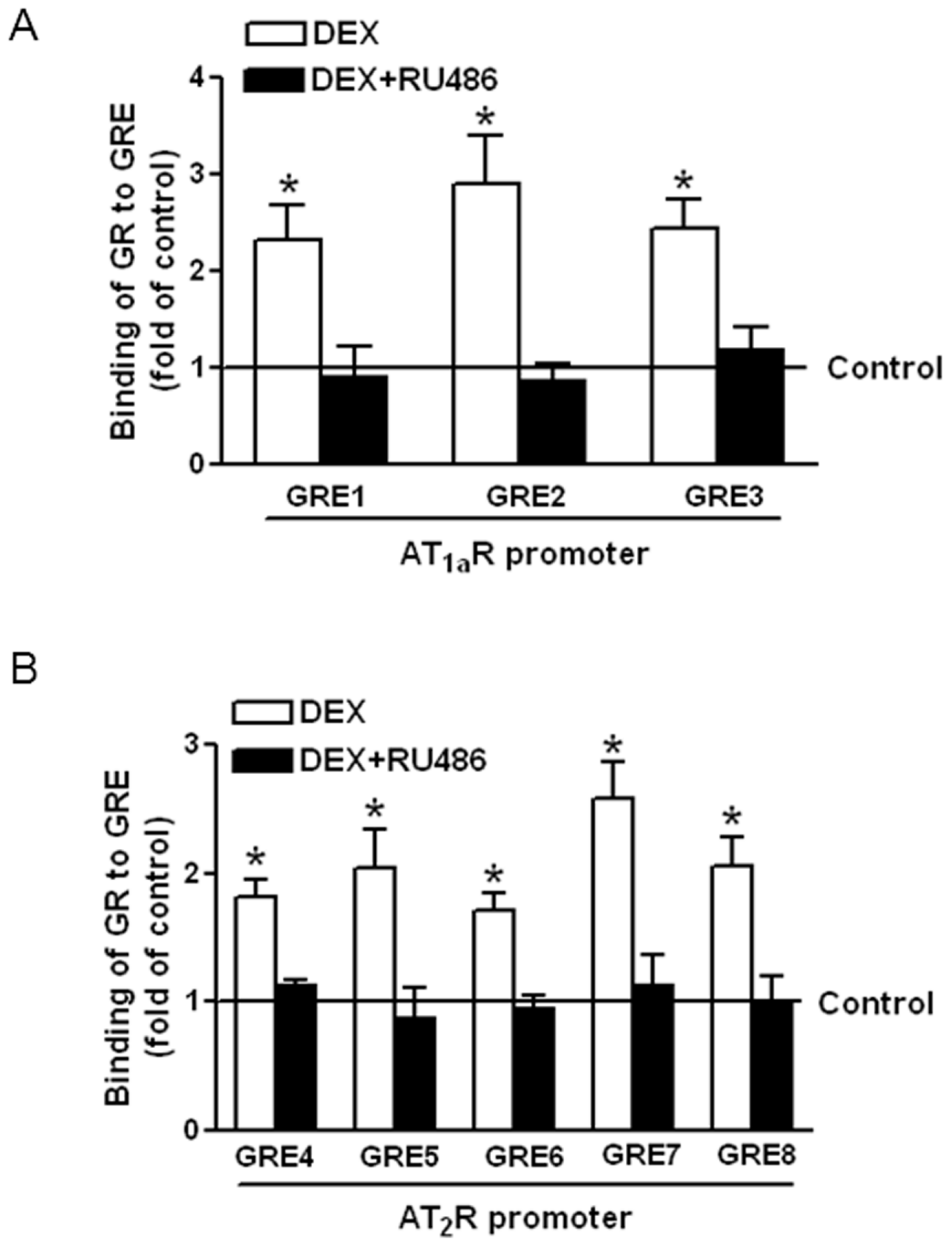
Effects of dexamethasone on GR binding to GREs at the AT_1a_R and AT_2_R promoters. Hearts were isolated from animals treated with vehicle control, dexamethasone (DEX), DEX + RU486. Binding of the GR to GREs at the AT_1a_R (**A**) and AT_2_R (**B**) promoters was determined by ChIP assays using a GR antibody. Data are means ± SEM. Data were analyzed by one-way ANOVA. * P<0.05, DEX *vs*. control or DEX+RU486, n = 5.

Dexamethasone treatment decreased total GR protein abundance but significantly increased nuclear accumulation of GR in the heart (Figure 4). RU486 restored GR levels in myocardial tissue to the control values (Figure 4). Previous studies have identified three GRE sites located in the proximal promoter of AT_1a_R gene, which positively regulate the gene activity [18]. In contrast, we have recently identified several GRE sites that suppress promoter activity of AT_2_R [20]. To determine the effect of dexamethasone on GR binding affinity to GRE sites, competition studies of electrophoretic mobility shift assay was performed in pooled nuclear extracts from the hearts with the increasing ratio of unlabeled/labeled oligonucleotides encompassing GRE2 at the AT_1a_R promoter and GRE4 at the AT_2_R promoter. Figure 5 shows that dexamethasone significantly increased the binding affinity of nuclear extracts to GRE sites at the promoters of both AT_1a_R and AT_2_R, which was blocked by RU486. To further determine the effect of dexamethasone treatment on GR binding to GRE sites at ATR promoters *in vivo* in the context of intact chromatin, ChIP assays were performed. Figure 6 demonstrates that dexamethasone significantly increased GR binding to multiple GRE sites at AT_1a_R (GRE 1, 2 & 3) and AT_2_R (GRE 4, 5, 6, 7, & 8) promoters in the heart. This effect of dexamethasone was blocked by RU486 (Figure 6).

### 4. Dexamethasone up-regulated PKCε expression and activity in the heart

**Figure 7:**
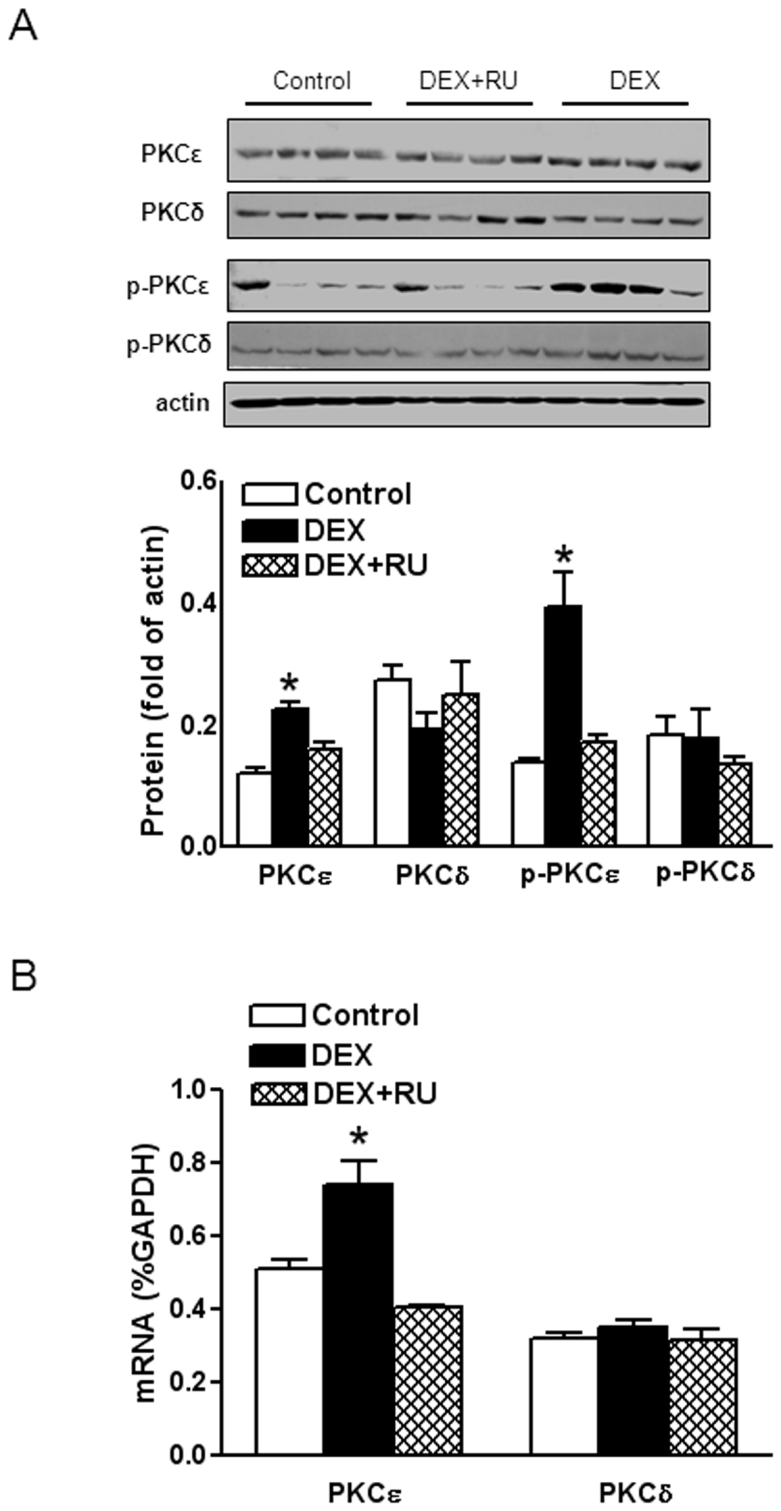
Effects of dexamethasone on PKCε and PKCδ expression. Hearts were isolated from animals treated with vehicle control, dexamethasone (DEX), DEX + RU486 (RU). PKCε, PKCδ, p-PKCε, and p-PKCδ protein abundance (**A**) and PKCε and PKCδ mRNA abundance (**B**) were determined by Western blots and real time RT-PCR, respectively. Data are means ± SEM. Data were analyzed by one-way ANOVA. * P<0.05, DEX *vs*. control or DEX+RU486, n = 4.

Given that angiotensin II receptors exert a regulatory effect on PKCε expression and activity, and that PKCε plays a pivotal role of cardioprotection in the setting of heart ischemia and reperfusion injury, we further determined whether dexamethasone treatment altered PKCε expression and activity in the heart. As shown in Figure 7, dexamethasone treatment significantly increased PKCε mRNA and protein abundance in the left ventricle. This was accompanied by a significant increase in the active form of p-PKCε levels in the heart (Figure 7). These effects were blocked by RU486 (Figure 7). Unlike PKCε, dexamethasone treatment had no significant effect on PKCδ expression and activity in the heart (Figure 7).

## Discussion

This is the first study to our knowledge revealing that short-term *in vivo* treatment with dexamethasone reduced AT_2_R and increased AT_1_R expression in the heart, which contributed to the cardioprotective effects of glucocorticoids in a setting of acute ischemia and reperfusion injury. The dexamethasone-mediated regulation of ATR expression and cardioprotection were through a GR dependent mechanism by increasing the binding of GR to GRE sites at both AT_1a_R and AT_2_R promoters.

The present study demonstrated that intraperitoneal injection of 1 mg/kg/day dexamethasone for 5 days reduced myocardial injury and improved functional recovery in a setting of acute ischemia and reperfusion insults. Clinically, dexamethasone is used in a wide range of doses from 0.4 mg/day to 40 mg/day in adults as well as from 0.15 mg/kg/day to 2 mg/kg/day in infants and children, given as a single injection or daily treatment up to 1 month. Based on body surface area, the dose of dexamethasone used in the present study is well within the dose range used in humans. This effect was blocked by a GR antagonist RU486 and, thus indicated a GR-dependent mechanism. Consistent with our findings, rats pretreated intraperitoneally with 2 mg/kg dexamethasone 24 hours before ischemia and reperfusion insults preserved myocardial function with significantly less frequent occurrence of ventricular arrthymias [8]. Additional studies have found that 40 mg/kg of methlyprednisolone 24 hours and 5 days before ischemia and reperfusion insults protected heart function and reduced myocardial tissue damage [7], [23]. Additionally, studies in male Wistar rats demonstrated that pretreatment with 3 mg/kg/day of dexamethasone for 10 days or the addition of dexamethasone to perfusate 10 minutes before and after ischemic insult was cardioprotective [9]. In contrast, male rats given 0.35 mg/kg/day dexamethasone for 15 days resulted in cardiac remodeling and dysfunction [24]. These studies suggest that the cardiac effects of glucocorticoids are context-specific and may depend on the dose and duration of glucocorticoid exposures. Perhaps short-term and high dose of glucocorticoids are cardioprotective, albeit long-term and low dose of glucocorticoid exposure may cause quite different consequences of deleterious cardiac function. Prolonged glucocorticoid exposure is thought to increase systolic blood pressure and induce metabolic dysfunction [6], which may cause myocardial remodeling and impair the heart from optimal recovery from ischemic insults.

The present study provides novel evidence that glucocorticoids induce cardioprotection in a setting of acute ischemia and reperfusion insults through the regulation of angiotensin II receptor expression patterns in the heart. We demonstrated that short-term *in vivo* dexamethasone treatment increased AT_1_R expression and decreased AT_2_R expression in the heart. Although the potential systemic effects of dexamethasone may not be excluded in the present study, a recent study provided clear evidence that intraperitoneal injection of dexamethasone and GR-mediated cardioprotective effect is local and the heart-specific, and the heart-specific function of glucocorticoid/GR is mediated mainly by the direct effect on cardiomyocytes rather than vasculature [25]. In addition, our previous study demonstrated that dexamethasone had a direct effect in down-regulating AT_2_R expression in isolated fetal rat hearts, which was blocked by RU486 [20]. In the present study, the finding that blockade of AT_1_R and AT_2_R with losartan and PD123319 abrogated dexamethasone-induced improvement of ischemia and reperfusion heart injury demonstrates the functional significance and cause-and-effect relation between angiotensin II receptors and glucocorticoid-mediated cardioprotection. In rats, blockade of AT_2_R increased myocardial protection from ischemia reperfusion injury while AT_1_R inhibition produces the opposite effect [20], [26], [27], suggesting that the ratio of AT_2_R to AT_1_R is an especially important consideration in cardiac susceptibility to acute ischemia and reperfusion injury. Thus, the pathophysiological function of angiotensin II receptors is context-specific, *i.e.* the ratio of AT_1_R to AT_2_R [15], [28]. In the heart, an increase in the ratio of AT_1_R to AT_2_R may be protective in a setting of acute ischemia and reperfusion injury, whereas the decreased ratio may lead to consequences of deleterious cardiac function. Indeed, it has been found that AT_2_R expression remains normal or increased in failing hearts while AT_1_R expression declines [29]–[31]. In present study, our findings that losartan but not PD123319 treatment abrogated dexamethasone-induced protective effect of heart ischemia/reperfusion injury suggest a predominant role of AT_1_R activation in glucocorticoid-mediated cardioprotection.

Unlike large mammals including humans in which only one type of AT_1_R is identified, rodents have two subtypes of AT_1_R, AT_1a_R and AT_1b_R coding from two separate genes. Whereas AT_1a_R is a human equivalent AT_1_R, AT_1a_R and AT_1b_R couple to similar signaling pathways and their physiological roles are similar and not clearly distinguished. Multiple GREs are present at angiotensin II receptor promoters in rodents. Glucocorticoids are known to promote transcriptional activity of AT_1a_R and the study of promoter/luciferase reporter gene constructs identifies GRE2 (−756 to −770) on the AT_1a_R promoter as being responsive to dexamethasone stimulation [18]. Our recent study demonstrated multiple GREs on the AT_2_R promoter that exert an inhibitory effect on the gene activities [20]. Site-specific deletion of each GRE independently caused a significant increase in the AT_2_R promoter activity [20]. In the present study, we demonstrated that dexamethasone treatment increased GR nuclear translocation and GR binding to GREs at AT_1a_R and AT_2_R promoters in the heart *in vivo* in the context of intact chromatin, suggesting a specific effect of glucocorticoid treatment on AT_1_R and AT_2_R gene transcription in the heart. The functional significance of this GR-mediated transcriptional regulation is further demonstrated by the finding that dexamethasone treatment increased AT_1a_R mRNA and AT_1_R protein expression and decreased AT_2_R mRNA and protein expression in the heart. The finding that RU486 inhibited dexamethasone-induced effects on transcriptional regulation and AT_1_R and AT_2_R expression in the heart is consistent with the previous finding of the direct effect of RU486 in inhibiting dexamethasone-mediated suppression of AT_2_R in isolated hearts [20], supporting the notion of a direct GR-dependent mechanism. Although changes in Ang II levels may contribute to cardiac pathophysiology, recent studies have demonstrated that alteration of Ang II receptor expression without changes in Ang II in stressed hearts plays an important role in regulating cardiac function [32], [33]. While it is not clear whether ischemia/reperfusion increases Ang II expression and/or release locally in the isolated heart in a Langendorff preparation in the present study, the findings that dexamethasone treatment significantly increased AT_1_R abundance in the heart and blockade of AT_1_R by losartan abrogated dexamethasone-induced protective effect, suggest an important role of increased AT_1_R expression in the glucocorticoid-mediated cardioprotection.

The finding of increased PKCε expression and the active form of p-PKCε in the heart of dexamethasone-treated animals is intriguing and suggests a possible mechanism in the cardioprotection observed. Angiotensin II receptors exert a regulatory effect on PKCε expression and activity. Thus, blockade of AT_2_R with PD123319 increased PKCε expression and AT_1_R stimulation and AT_2_R inhibition mimic ischemic preconditioning by increasing PKCε activity [34]–[36]. In the present study, we demonstrated that dexamethasone treatment significantly increased PKCε mRNA and protein expression, as well as increased the active form of p-PKCε in the heart in a GR-dependent manner. Whereas whether this GR-induced increase in PKCε expression and activity in the heart was mediated by angiotensin II receptors remains to be determined, that dexamethasone treatment up-regulated PKCε expression and activity has been demonstrated in porcine coronary arteries [37]. It has been well documented that PKCε plays an important role in ischemic preconditioning and is necessary and sufficient for cardioprotective effects in ischemia-reperfusion injury [38]–[40]. Interestingly, it is noted that dexamethasone treatment resembles the effect of cardiac preconditioning [41], [42]. The finding that dexamethasone had no significant effect on PKCδ expression and activity in the heart suggests that its effect on PKCε in isozyme-selective. Studies with transgenic mice have shown that activation of PKCδ increases injury from ischemia reperfusion and inhibition of PKCδ exerts protective effect [43]. These findings suggest that dexamethasone promotes cardioprotection through modulation of the relative ratio of PKCε to PKCδ in the heart. In addition to PKC, Tokudome et al [25] observed that glucocorticoid protected hearts from ischemia reperfusion injury by activating cyclooxygenase-2 (COX-2) and prostaglandin D biosynthesis. The previous study has also shown that COX-2 expression is differentially regulated by AT_1_R and AT_2_R [44]. Perhaps glucocorticoids modulate the expression pattern of angiotensin II receptors in the heart, which produces an intracellular environment conducive for cardioprotective factors such as PKCε and COX-2 to predominate, resulting in increased protection of the heart against ischemia and reperfusion injury.

## Conclusions

The present study provides novel insight concerning the regulation of AT_1_R and AT_2_R in the heart following glucocorticoid treatment and demonstrates a mechanism by which short-term glucocorticoid treatment improves myocardial recovery in a setting of acute ischemia and reperfusion injury. The vital role of GR in glucocorticoid-mediated cardioprotection is indicated at the molecular level. Given the myocardial protection afforded by short-term use of glucocorticoids, there is potentially clinical relevance in the glucocorticoid treatment in limiting myocardial dysfunction and improving mortality following invasive cardiac procedures, or major surgical operations where increased myocardial strain is common.
